# Abstracts and Gleanings

**Published:** 1884-05-20

**Authors:** 


### ABSTRACTS AND GLEANINGS.

**California Laurel**.—Dr. Mann-Hammond, M. D., Kansas City, Mo., in Therapeutic Gazette, writes :

I regard the California laurel as a very valuable addition to the materia medica, although I have not seen much written upon it. But we are all slow to write what we know. We have pondrous volumes of theories and little primers of facts. I sometimes think the unwritten knowledge of medicine exceeds the written. Several of my medical confreres have, to my knowledge, been using this drug with marked success; but they are busy practitioners, who never write.

At the time I first employed the laurel, I was living in California. I made a number of experiments with the crude material, in rheumatism and nervous diseases. After the drug had been prepared as a fluid extract, Dr. Bundy, that indefatigable student, to whom we owe much of the knowledge of the remedies of the Pacific slope, in an article to the Therapeutic Gazette corroborated my experience, and added testimony to the value of the laurel in cholera morbus, dysentery, diarrhœa and summer complaints; also in convalescence from typhoid fever.

Since then I have employed it in so many cases of cholera morbus and dysentery that I could not enumerate them; and with such marked success in all, that it has come to be the first remedy thought of by me in all those sudden seizures incident to hot weather, the use of ice water, green fruit, etc.

Its action cannot properly be called an astringent. It is, rather, stimulant and anæsthetic. I give three drops of the fluid extract on sugar, dissolved in a little water, every fifteen minutes or half hour, and changing to an hour between doses when relief has been obtained. In a case of a lady in charge of another physician, dysentery had continued for three days with spasmodic abdominal pain, which the usual remedies had not relieved. I was sent for in the night, and after the second dose of California laurel had been taken the patient fell asleep.

It will shorten the congestive or chilly stage of chills and fever. I was having chills and fever, and during one of the chills the pain was severe, from the intense congestion, and I asksd to have a dose of California laurel administered. It gave such marked relief that I had the dose repeated in a short time. The congestion was completely relieved, a profuse perspiration set in, and, in short, the chill was broken. During the apyrexia I took quinine, so I cannot say whether the laurel has antiperiodic properties. But the relief it gave during the paroxysm and its shortening of the period of congestion gives promise of its being a remedy worthy of study in that connection.

California laurel is a great pain obtunder, with no narcotic or cumulative effect. Here let me say that the dose which I first recommended, and which Messrs. Parke, Davis & Co., have put on their labels, viz., ten to thirty drops, I think too large. I now use from three to ten drops at a dose. In making a prescription, glycerine or simple syrup should be used as a vehicle.

In conclusion, I will say, I have used this drug in rheumatism, in all forms of neuralgia, nervous headache, cerebo-spinal meningitis, cholera morbus, cholera infantum, diarrhœa, dysentery, bilious colic, dysmenorrhœa, until I could write a monogram on each disease, made up of cases in which California laurel was the chief remedial agent.

Physicians should use California laurel more freely than they do, and freely report their experience, in order that the virtues and peculiarities of this valuable drug may be thoroughly canvassed. I most respectfully request that any physician who has used it at all, please drop me a postal card giving his name and address.

1307 Walnut St.

**The Bromide of Sodium vs. Like Salts of Potassium.**— In an article in the Lancet of December 22d, 1883, T. J. Hudson, M. B., L. R. C. P., London, discusses the comparative merits and demerits of the bromide of iodide of sodium and potassium. He concludes from physiological experiments and clinical results that the sodium salts are to be preferred to those of potassium when it is desired through them to secure the characteristic medicinal properties of bromine and iodine on the system.

He proceeds to give the results of his clinical experience in the use of these two salts at the Leeds Public Dispensary. In pertussis, eighty-seven cases of which were treated, he found the best results from the bromide of potassium, owing to its greater depressant action on the cerebrum, etc., and should give it in preference, unless great weakness already exists, as in rickety children and others. In women approaching the menopause and suffering from the usual train of nervous symptoms, with abdominal and cranial pains, tinnitus aurium and flatulent dyspepsia, the sodium salt in half-drachm doses causes less depression and sinking than the potassium salt, and the dyspepsia is relieved and soon cured. In the weary muscular pains following severe straining, as after diarrhœa, the sodium salt is of great comfort, and brings on no return of the looseness, as the potash salt often does. In the various forms of headache, neuralgia and nervous excitement where a hypnotic is needed, the sodium salt in large doses is followed by little or no sign of lassitude, nor signs of weakness, as is often seen after potash. This applies also to insomnia from whatever cause arising, and to puerperal mania with great depression. Moreover, the hypnotic action not being so great as that of the bromide of potassium a larger dose (one-fifth) is always given in these cases. It is also useful in incipient and pronounced delirium tremens, also in hysteria and chorea, and is longer than potassium salt in making a decided impressien when pushed in these cases. In severe cardiac cases, where pain and insomnia threaten to bring on dementia, and also in cardiac cases complicated with epilepsy, where a depressant and toxic action of the potassium salt is to be feared, fifty to eighty grains of sodium bromide may be given without affecting the heart muscle to any extent, this being given for many nights.

In the irritable and palpitating heart without organic lesion, as in excessive tea and coffee drinking, and when of dyspeptic origin, the bromide of potassium is to be preferred. In epilepsy the sodium salt may be administered from eight to ten months, in his experience, with far less toxic signs, or those of depression, than complicate the action of the bromide of potassium.—*Therapeutic Gazette.*

**Tonga.**—In an obstinate case of neuralgia Dr. Oak, (in Therapeutic Gazette) says :

I had thought of the new remedy "tonga" several times during my experience with this case, but as there was none in the drug store in this place, did not have a chance to try it. As the case was growing worse all the time I secured a supply from a neighboring town, of which I gave a teaspoonful every three hours. By the time the third dose was taken the pain was all gone. Ordered the medicine continued in teaspoonful doses, three times a day until all was taken. About three weeks after the medicine gave out, the neuralgia returned. A return to tonga caused it to promptly disappear.

Case 2. F. M., æt, about 30 years. Neuralgia of the supraorbital branch of the fifth pair on the left side. The pain in this case was intense. Had not slept any during the night previous to my call. Left eye swollen, and inflamed. Prescribed an ounce of fld. ext. tonga, to be taken in teaspoonful doses until relieved. Saw him on the street next day when he told me that by the time the third dose was taken all pain had disappeared.

**Tincture of Gelsemium Hypodermically in Convulsions.**—A writer (in American Medical Journal), reports a case of convulsions in a child of eighteen months of age, who had fallen twenty feet, striking the head upon a brick pavement. The child was picked up limp and unconscious; for twelve hours it lay in continuous spasm, with insensibility, jerking of the facial muscles, arms and fingers. Eight drops of the green root tincture of gelsemium was then administered hypodermically into the thigh. In fifteen minutes all spasms ceased, and the child rested quietly for six hours. At the expiration of this time the covulsions again returned and continued without abatement for three hours,at which time I arrived and repeated the injection as before.

In a few moments the spasms again ceased and the child rested quietly. After the second injection there was no further trouble in administering medicine per orem, and recovery was finally complete.

The action of gelsemium upon the nervous system is marked. In large doses it produces giddiness, dilated pupil. and sometimes a peculiar swimming of the head. It is a powerful relaxant to the muscular system, and it diminishes rapidly the circulation; for this last property it is highly esteemed in hyperæmia of organs, especially of the brain and uterus. It is a valuable arterial sedative, controlling the circulation by its influence upon the vaso-motor nerves. In convulsions due to excess of blood in the medulla oblongata or pons varolii, or in congested states of the uterus, the hypodermic use of gelsemium has proved highly satisfactory.

I do not hesitate to administer large doses in such cases. From half to one drachm of the green root tincture in adult cases—doses sufficiently large to produce complete muscular relaxation. I have never had any bad results following, no abscess, not even redness at the point of insertion.

I am pleased to know that we have an agent that can be used hypodermically with such comparative safety, so potent in its action, and an agent that will bring the answer so surely.

**Chancres and old Sores.**—Soft and hard chancres are successfully treated by applying the following mixture :

℞ Salicylic acid, iodoform......................aa ℥ss,
Cannabis indica (solid).......................gr- x,
Surgical collodion............................℥ j.

M. Collodion will dissolve salicylic acid readily, and iodoform slowly. In using this, shake well and apply with a small brush, immediately to the chancre or sore. It dries immediately, and completely protects the part. This will effectually destroy the specific character of the sore, and a kindly action, healing process, is set up at once. A slight burning sensation is experienced when we make the application, but this is soon followed by a cool, agreeable sensation; and when we examine the part carefully, and come to handle it, we find that a condition of partial anæsthesia exists. The application should be repeated as often as it is thrown off, which will be every two or three days. This is not only a convenient plan of treatment, but it is generally quite successful. Where the ulcers do not heal readily under this treatment, we may omit for a few days and dress with Mayer's ointment.—*American Medical Journal.*

**Incubation of Infants.**—The Medical Press, February 27, 1884, says :

The results obtained by the aid of an incubator for rearing infants have just been published by M. Auvard, resident at the Maternity Hospital, in his Archives de Tocologie. The first incubator was used in Paris by M. Tarnier, and consists of a large wooden case divided into two portions, the lower of which containg warm water, while the infant is placed in the upper one.

At first the incubator was heated by means of a thermo-syphon, but this system has been abandoned, fearing that through the negligence of the attendants the lamp would be left alight too long. The apparatus is now heated by filling the reservoir witn boiling water both morning and evening, and thus a mean temperature of 30–32° C. is kept up. The child is clothed in the usual garments, because, if the skin was in contact with the air at a temperature of 32° C., it would lose some of the animal heat, and when taking out the infant to feed it, the danger of cold would be very great were it unclothed. Among the different cases (151 in number) in which the incubator was used, there were 93 infants which were born prematurely. Of these 31 died. M. Auvard based his statistics on the weight of the children, and discovered that among those who weighed less than 2,000 grammes the death-rate was at the "Maternite," 66 per cent.; at that of Paris, 65 per cent.; while in those places where the incubator was used, it was only 38 per cent. In the treatment of œdema, which is a serious infiltration of the tissues caused by the bad state of the infant when born, and the rigors of the exterior temperature, and in which, as a rule, 16 out of every 20 cases die; when the incubator was employed, only 4 of the 25 placed in it failed to recover. It has has been said that children brought up in this way die when exposed to the outer air, but such is not the case, as M. Auvard conclusively proves. It has also been stated that immature children placed in the incubator only live one or two months, viz : until the time arrives in which they ought to have come into the world; but this objection is not founded on any scientific basis, and is therefore not of any value. M. Winckel, of Dresden, has started a new theory of permanent baths, but we have not investigated it sufficiently to form any opinion.—*Med. and Surg. Reporter.*

**A New Method of Removing Nasal Polypi.**—In the Canada Medical Record, February, 1884, Dr. William Ralph Bell says :

"I take the liberty of bringing the mode of treatment before the notice of your readers, which I have practiced with the very best results in several cases. It obviates any trouble from hemorrhage, which is frequently the case when the forceps or hook is used; it is painless and very simple. I get my patient to blow strongly through the effected nostril, closing the other with his finger. The polypus will be brought down so that it can be easily seen through the external nares; then with my hypodermic syringe charged with a solution of tannic acid in water (of the strength of twenty grains to the fluid drachm), I pierce the polypus with the needle, and inject ten, fifteen or twenty minims of solution, according to size of tumor. In a few days the polypus shrivels and dries up (tanned); it comes away without any trouble or pain, and looks ike a clot of dry blood, my patients usually removimg it by blowing the nose or by their fingers. In only one case, that of an old lady, had I occasion to remove it myself, and in her case I think she was afraid to do so, for when I seized it wifh a pair of dressing forceps, I required to make no traction to bring it away."—*Med. and Surg. Reporter.*

**How to Take a Pill.**—With many patients the swallowing of a pill is a very difficult matter. Why this should be so is somewhat difficult of explanation, inasmuch as these same patients will readily swallow a pea of the same size as the sugar-coated pill, in the deglutition of which they experience so much difficulty. Dr. Wills suggests a method in the Medical and Surgical Reporter, which he says he has used for several years with success, and which may be new to many of our readers. Having noticed that if a person at meals inclined the head backwards, as in laughing, while there was food in the mouth, he was pretty sure to be strangled from the "food going the wrong way," Dr. Wills instructed those of his patients who had difficulty in swallowing pills to keep the head in the position it would occupy if they were eating and swallowing food at the table—that is, the head inclined forward and the chin near the breast, and kept in that position. If a small portion of saliva be on hand, or a small quantity of water taken after the pill is taken into the mouth, it will surprise the patient and gratify the doctor, he says, to witness the facility with which it will be swallowed. He has found that to direct the patient to keep his eyes on his toes while swallowing, will succeed in keeping him from throwing his head back.—*The Therapeutic Gazette.*

**Bromide of Arsenic in Diabetes.**—The Journal de Medicine et de Pharmacie de l' Algeri tells us that this drug has been employed four times successfully by Dr. Arpad Bokai. He prepares as follows:

Mix the following in a glass tube:

| | |
|---|---|
| Arsenious acid / Carbonate of potassa } | ..................aa gr. j. |
| Distilled water... | ......................5 drops. |

Heat to limpidity and add enough distilled water to make 10 grammes. Add four drops of bromine and let the mixture stand for a day, when it is ready for use.

A diabetic, 22 years of age, who at the time of his admission to the clinic, could hatdly mount the staircase, was entirely relieved in a little less than three months. In that interval he gained in weight 8 k., 100 gr., and the proportion of glucose, which had varied between 170 and 111 grammes a day, was reduced to 0. During the last days the medicine was suspended and the patient nourished with rice.

The patient was submitted at first to a strict diet for 17 days, which diminished the proportion of sugar to about one-half. He then received the bromide for 11 days in three-drop doses, which was increased to 6 drops a day, in consequence of which the proportion of sugar diminished rapidly, and the analysis gave only traces. After introducing certain modifications to the diet, a small quantity of sugar was noted in consequence for 27 days Five drops of the bromide was given, and 150 grs. of rice added to the diet list. Finally the drug was abandoned, the diet restrictions removed, and the patient discharged relieved.—*In American Medical Association.*

**Investigations into the Physiological and Therapeutic Properties of Trinotrine.**—M. Marieux has studied the effects of this drug, (Bull. et Memoirs de la Soc. de Therapeutique) in 23 experiments made on guinea-pigs, frogs, rabbits and dogs, and on man These experiments were made in the presence of M. Dujardin-Beaumetz, and with the assistance of MM. Eloy and Huchard, with the following conclusions:

1. Trinitrine acts on the brain by producing phenomena of congestion.

2. It accelerates the heart beat, dilates the peripheral vessels, and diminishes arterial tension.

3. This vascular dilatation seems to depend upon a paralysis of the great sympathetic.

4. It acts but feebly upon the respiration, which becomes irregular.

5. It has but little effect upon the secretions.

In toxic doses :—

1. It produces, in the lower animals, clonic and toxic convulsions.

2. With the rabbit and dog, the toxic dose is variable, but is very large in all cases.

3, In man it commences with 10 drop doses of the alcoholic solution.

In the therapeutic application :—

1. It is indicated in all cases where cerebral anæma might lead to fatal results.

2. It gives good results in rebellious neuralgias of anæmic origin.

3. Its most marked action is in angina pectoris.

4. It is of no use in diseases of the kidneys or lungs.

5. It is contra indicated in all affections where there exists active or passive cerebral congestion.

6. It can be administered very satisfactorily in 4 to 6 deop doses of the alcoholic solution of 1 to 100, in the 24 hours, either as a potion or in hypodermic injections.

7. When compared to the action of nitrite of amyl, it is less prompt and less sure, but its effects are more enduring. In many cases it would be well to combine the two—*In American Medical Journal.*

**Mastitis.**—The situation as regards broken breasts may be summarized thus: 1. The doctor is often not called until thirty-six or forty-eight hours after inflammatory trouble has commenced. 2. He does not know what to do after he is called. 3. He poultices the breast too much, all treatment being local and not affecting materially anything but the skin. Now if this were different and the doctor enquired after the breast three or four days after delivery, and the moment the patient complained of soreness about the nipples order a shield and tube for the infant, and at once apply adhesive plasters in such a way as to relieve the gland from the tension of its own weight, inflammation and supuration might be prevented. If, however, the soreness has existed three or four days and the patient has chilly sensations along her back and there are hard painful lumps in the glands, he may accomplish good by resorting to the afore-mentioned methods, and, in addition, thrusting a very sharp bistoury into and through the indurated lumps. If pus is found the incision may be enlarged to admit good drainage as the bistoury is withdrawn; if not the resulting hemorrhage will generally relieve the vascular engorgement. The bowels should be opened by a saline preceded by a mild mercurial, say 10 gr. of calomel in two doses, three hours apart. Opium should then be given for the same reason that it is given in other inflammatory affections. When suppuration has occurred before the doctor is called, free incision of the abscess should be performed and the wound dressed with absorbent cotton moistened with a solution of corrosive sublimate, one grain to the pint of water. It is better to make free openings with the knife than to annoy the patient by deluging putrefying abscesses with antiseptic solutions. The daily use of a syringe for this purpose irritates the patient more than a good free cut made but once.

The aim should be to give the gland as much physiological rest as possible, and while belladonna may be able to arrest grandular activity in a physiological sense, practically it can do nothing of the kind while the baby is tugging at the other breast or while the maternal impulses are encouraging the gland-cells to make the most of the blood that is being supplied to them. It is better to look at the matter as it is, and not be satisfied with treatment which is theoretically sound, but has been found in practice of no use. The firm and uniform pressure imposed by properly applied adhesive plasters does more to accomplish physislogical rest for the mammary gland than any drug that has heretofore been employed.—*The Medical Age.*

**Experiments With the Sputa of Phthisical Patients.**—M. Vignal has been trying some experiments with the view of ascertaining whether the sputa of phthisical patients, as found in the streets, still contained bacilli. He collected a certain quantity of such sputa, and submitted it to desiccation : he then moistened it, and let it dry again at different times, so as to place it as much as possible in the condition in which it would be found in ordinary circumstances in a room. He discovered that sputa thus treated contained bacilli as numerous and as well formed as if they had just been expectorated. He inoculated two guinea-pigs with the matter; one of which died in a few days, from obstruction in the bowels, and he could not, in consequence, come to any conclusion. But the second animal, though it increased in weight during the first few weeks subsequent to the injection, afterwards began to lose flesh, and died in about three months. At the autopsy it was found that in all the organs there were a great number of tubercles which contained bacilli. M. Vignal concludes that sputa of phthisical patients, as found on the ground, in the streets, or in apartments, are far from being inoffensive, and might become agents of contagion to persons predisposed, or in whom the bacilli would find a favorable soil for propagation.—*Pop. Science News.*

**Common Sense in the Lying-In Room.**—The following from an old veteran, who expresses himself in a letter to the Medical Record, sounds a little odd at this particular time, so soon after the discussion before the New York Academy of Medicine. It will, nevertheless, strike many as being very sensible : "My boy," says the old sage, "I am an old man; I have practiced over fifty years, and, as you are aware, have been very successful. In regard to your labor cases, I would give you the following advice : "First, as you intend practicing in the city, make a habit of leaving your forceps at your office; for if you ever require them you will always have ample time to send for them, and your not having them with you may save some woman much trouble. Second, do not examine your patient much or often. See that things are right, and then let nature manage the case. Third, instruct your patient when she desires to empty bladder or rectum, to have her night-glass conveniently placed, near the bed in a chair, and, while supporting the abdomen with her hands, to rise carefully from her bed to the stool; and to return in the same way. Her rising will allow all clots, fragments of placenta, etc., to pass from the vagina, and you will be surprised to see how few cases of puerperal fever you will be troubled with. As for its bringing on hemorrhages, that is all bosh. Let your patients keep the outside of their bodies clean, attend to the calls of nature in a common sense way, and depend upon nature to keep the inside all right, and you will be surprised at your success in this line of practice."—*The Medical Age.*

**Santonine.**—We usually think of santonine as a vermifuge only, in which it stands at the head of its class; but it has other important properties. I will not tarry now to discuss how or why it has a peculiar influence over the bladder, which renders it so efficient in overcoming, in some special cases, that severe burning and scalding sensation and tenesmus of the bladder, but only stop to say, in addition, that in some cases of retention of urine, a few small doses of santonine will prove to be the remedy par excellence.

HYPOSULPHITE OF SODA.—Last but not least, I wish to notice briefly hyposulphite of soda.

Standing as it does in the list of alkalies, and fulfilling their general indication, yet it seems to subserve a special purpose of its own. If we have acid fermentation in the stomach, indicated by acid eructions, coated tongue, or rather furred with a white or greyish-white or dirty color, accompanied, in children especially, with colic and green acrid discharges of the bowels, we naturally think of alkalies. If our patient is suffering with boils or abscesses of the cellular or muscular tissue, we say lime is the remedy, as it is the salt which preserves these tissues; or, if the coating of the tongue is a clean white, in the absence of any destruction of tissue, we use bicarbonate of soda, believing that through its influence on the blood it influences nutrition as well as antidotes the acid; but when we have the dirty gray or brown color, tongue pallid and broad, accompanied with foul breath and fever, then the antizymotic influence of hyposulphite of soda will correct all, and lead our patient out into the sunlight of health and happiness.—*Amer. Med. Digest.*

**The Best Time for Administering Medicines.**—Before or after meals? Such is the question often asked of the doctor, but the answer is not always ready. The Midland Medical Miscellany answers it as follows: Medicines that are irritating should be given after meals, when the stomach is full, viz.: the salts of copper, zinc, iron and arsenic, in large doses. Small doses, intended to act on the stomach terminals of the vagi, must be given when the organ is empty. Chemical reasons also have their influence: thus, oxide and nitrate of silver, intended for local action, should appear in the stomach during its period of inactivity, lest, at other times, chemical reactions destroy the special attributes for which these remedies are prescribed. Iodine and the iodides further illustrate this point. Given on an empty stomach they promptly diffuse into the blood, but if digestion is going on, the acids and starch form products of inferior activity, and thus the purpose which they were intended to subserve is defeated. Substances prescribed to have alvinel action on the mucous membrane, or for prompt diffusion unaltered, are preferably given before meals. The condition of the stomach veins after meals is such as to lessen the activity of diffusion of poisons, and hinders their passage through the liver. It follows that active medicaments in doses near the danger-line, are more safely administered after meals.

When shall acids and alkalies be given, before or after meals? First, as to acids. When acids are prescribed with the view to check the excessive formation of the acids of the gastric juice they may be given before meals—as, by the laws of osmosis, they will determine the glandular flow of the alkaline constituents of the blood. The same reasoning would hold good when the alkaline condition of the blood is in excess; osmosis being favored, the acid would reach the blood more readily. Second, as to alkalies. These may be given just before meals, when the acid forming materials in the blood diffuse into the stomach glands, and after digestion is completed, when the alkalies diffuse directly into the blood, without interference from the contents of the stomach. An alkali taken during the time when the reaction of the stomach juices should be strongly acid, must necessarily hinder, if not arrest, the digestive process for the time being. The metallic salts—notably corrosive sublimate, alcohol, tannin and some other agents—impair or destroy the ferment, or digestive power, of pepsin. Wine that is intended to act as a food, is most beneficial when taken slowly during the course of the meal. The objection as regards the ill-effect of alcohol on pepsin is not applicable here, except to the stronger spirituous wines in large quantities, for the ordinary medicinal wines do not have sufficient alcoholic strength to injure this ferment. Iron, phosphates, cod-liver oil, malt and similar agents should, as a rule, go with food through the digestive process, and with the products of digestion enter the blood.—*Med. Age.*

**The Bed Pan in Child-bed.**—"An Old Practitioner" (in Med. Age) remarks: "Great stress is laid by many on keeping the woman rigidly on her back for a week after delivery, and to that end the bed-pan is to be employed. The bed-pan is one of the devices of the devil and should be exorcised from the lying-in room with the arch-fiend who conceived it. Let the woman sit up when she desires to evacuate either rectum or bladder. Nature never designed that man, male or female, should lie down on his back to defecate or urinate, and, in the case of the lying-in woman, to have her assume this position for either of those acts, is to deprive her of one of Nature's means of prophylaxis against the dangers to which she is liable. Seat her on the vessel, and thus give her the combined assistance of gravitation and abdominal contraction in ridding the uterus and the vagina of the debris, which, undergoing decomposition, gives rise to the septic matter, which, when absorbed, gives us puerperal fever, or puerperal septicæmia—synonymous terms, in my opinion. The vaginal injections, the necessity of which is insisted on by some, I regard as a piece of meddlesomeness which should be mentioned only to be condemned. They not only disturb the woman, but they remove from the vagina the lochia with which a kindly and beneficent Nature has bathed the parts, as with a poultice, to soothe the injury she has done in the violence of her efforts to unload her precious cargo.

**Treatment of Placenta Prævia.**—He objects to the prevailing custom of rupturing the membranes; he, on the contrary, maintains their integrity as long as possible, and for that purpose *introduces acepticallv prepared sponges* into the uterine segment as a hemostatic. The advantages for both mother and child, are:

1. A uniform, harmless and simple appliance in all cases of placenta prævia (except in abortions up to four months).
2. Immediate and durable arrest of bleeding, while labors are being induced at the same time, until the os is amply dilated; prevention of post-partum hæmorrhages by the increase of uterine involution.
3. Preservation of the membranes until complete dilatation of the os tincæ; prevention of laceration of the cervix, in case turning should have to be resorted to; also, greater safety for the child.
4. Preservation and husbanding of the strength of the patient from the very beginning of this treatment; lessening the danger of a collapse, so often the result of former methods of treatment.—*Volkmann's Samuel Klin. Vortage.—St. Louis Medical and Surgical Journal.*

**Renal Calculus.**—Dr. Jourdan (in Philadelphia Medical and Surgical Reporter) writes: Mr. D. V. D., æt. 27, of billious temperament, began to complain of dull aching pain in the lumbar region in June, 1883. Being hard-worked mentally, and of a very sedentary habit, I attached little importance to his frequent, yea, almost constant complaints, till in October, when he was taken down with nephritic colic. This was relieved in the usual way with morphia hypodermically, etc. Contrary to the rule in a majority of these cases, the attacks recurred again and again. Confirmed, then, in the opinion that I had an impacted, or, at least, a calculus in the kidney to deal with, I gave him all the usually prescribed remedies for the solution of the stone. But my hopes were blighted every time, till, in January, 1884, I concluded to order Lithiated Hydrangea (Lambert & Co.). I prescribed it in drachm doses four times a day. The patient, who, by the way, is a stu dent of medicine and very intelligent, expressed himself in a few days as feeling better. He had only one attack of colic during the time he took the first bottle, and when he had used the second bottle he had a very light attack, in which he passed a skeleton-stone, *i. e.*, a mere shell. He is still using the medicine, but has had no return, and to all appearances is well. He used the Hydrangea in one other case with favorable results.

Well do I remember, some years ago, of attending on Mr. F., æt. 40 years, German descent, usually healthy, strong and robust, but then suffering with severe pleuro-pneumonia, and most intensely with the pleuritic stitch, which was so interfering with respiration as to be alarming at times; and after prescribing the usual sedatives, aconite and veratrum for fever, with full doses of Dover's powder and morphia to control the pain, and feeling confident of early relief, I repaired to the country. But some hours after my visit, instead of the expected relief, the pain in the chest became more severe and the interference with respiration more alarming, and another physician, my friend T. G. Matheny, was called to administer to him until my return. His prescription was tinct. bryonia and tinct. asclepias aa gtt., xx.; water ℥ iv. M. Sig. One teaspoonful every thirty minutes until pains were relieved, and every hour thereafter.—*Amer. Med. Digest*

**Lobelia.**—Let us hastily glance at this, another of the nauseant and emetic medicines, when given in full doses. Like its relative, ipecacuanha, its physiological is different from its drug effect. Given in cases of difficult or oppressed breathing, suffusion of the face, congestion, and especially in mucous rattling of the bronchial tubes, small doses of lobelia will improve innervation, give energy to the oppressed organs, and enable them to throw off the congestion and over-supply of mucous secretion; while in a little larger doses short of its emetic effect, it is an excellent antispasmodic in croup, asthma, and, in the hands of the obstetrician, proves a kind and valuable remedy in overcoming the rigidity of the undilutable os uteria, when given in one-drop doses, repeated every fifteen or twenty minutes.—*Amer. Med. Digest.*

A singular co-incidence connected with the death of Dr. Lunsford P. Yandell, was the following: On the day of its occurrence he related to a brother practitioner the case of a patient to whom he administered a dose of choral for the relief of a painful affection of the heart. It relieved him for a few moments, but he then fell back in his chair and died. Dr. Y., thought the death was too sudden to have been caused by the chloral. In describing the case he imitated the actions of the dying man, falling back in his chair and throwing his arms about. He was himself subject to angina pectoris and a few hours later had an attack for which he also took a dose of chloral, experiencing a momentary relief from pain, and then, falling back, his spirit went out.—*The Medical Age.*

**A Bird's Appetite.**—Dr. Wood says: "If a man could eat as much in proportion as a bird, he would consume a whole round of beef for his dinner. The redbreast is a most voracious bird. It has been calculated that, to keep a redbreast up to its normal weight, an amount of animal food is required daily equal to an earthworm fourteen feet in length. Taking a man of average weight, and measuring bulk for bulk with the redbreast, I tried to calculate how much food he would consume in twenty-four hours, if he ate as much in proportion as the bird. Assuming a sausage nine inches in circumference to be a fair equivalent of the earth-worm, I find that the man would have to eat sixty-seven feet of such sausage in every twenty-four hours. I mention this in order to illustrate the amount of work which is done by insect-eating birds.—*Pop. Sci. News.*

**The Simplest and Safest Method for the Rrdical Cure of Hemorrhoids.**—The London Med. Record, May 15, 1883, says that Scarenzio recommends (Renaiconti del Regie 1st Lomb., 1882,) the elastic ligature in preference to all other and more complicated methods, as being the safest and most effectual for the removal and radical cure of hemorrhoids. Its gradual action allows time for the formation of a firm clot in the veins, and the mass separates after two or three days, leaving a simple citatrized wound. The pain caused is trifling, and only lasts a short time. he has operated often by this method, and has never seen any bad effects follow.—*Med. & Surg. Reporter.*

**Remarkable Recovery.**—Dr. J. C. McMillan, in N. Y. Med. Record, reports the history of a man, an engineer, who had received a cut in the abdomen during a fracas. The cut was 4 inches long, near the umbilicus, and extended into the peritoneal cavity. The intestines were protruded, and the small intestine cut half through in one place. The patient had lain in the road for four hours before Dr. McMillan saw him, and the protruded bowels were covered with dirt. He was taken to a house, anæsthetized, the intestines cleaned with luke-warm water, the small intestine sewn up with silk suture, and the abdominal wound closed. The patient was well and discharged in two weeks.

**Digitalis as an Anaphrodisiac.**—Dr. N. L. Folsom reports in the Medical World on the effects of digitalis in the allaying of sexual appetite. Administered in ten-drop doses, three times a day, he declares that it will effectually overcome the imperious passion. It may be necessary to increase the dose to a teaspoonful in some cases. If it shall do in the hands of others what Dr. F. claims for it, the discovery will prove to have been an important one.—*Med. Age.*

"Doctor," said the grateful patient, seizing the physician's hand, "I shall never forget that I owe you my life." "You exaggerate," mildly replied the disciple of Æsculapius, "it would be exactly correct to say that you owe me for fifteen visits, and you will please not forget this fact."—*The Medical Age.*

